# How Do Women Vote: What Women Post About Home Birth Versus Hospital Birth on Popular Social Media Platforms

**DOI:** 10.7759/cureus.57621

**Published:** 2024-04-04

**Authors:** Kelsey Morris, Fiona Lane, Anita L Nelson, Hindi E Stohl

**Affiliations:** 1 Obstetrics and Gynecology, College of Osteopathic Medicine of the Pacific, Western University of Health Sciences, Pomona, USA; 2 Obstetrics and Gynecology, College of Osteopathic Medicine of the Pacific - Northwest, Western University of Health Sciences, Lebanon, USA; 3 Obstetrics and Gynecology, Harbor-UCLA Medical Center, David Geffen School of Medicine at UCLA, Torrance, USA

**Keywords:** social media, free birth, midwifery, birth, birth trauma, maternity care deserts, hospital birth, home birth

## Abstract

Introduction: The rates of home birth have been increasing; reliance on social media as a source of medical advice and support for patients has also been increasing. This is the first study that directly evaluates birthing people’s perceptions, attitudes, and advice about planned home births expressed in public posts and comments on two popular social media platforms - Reddit and TikTok.

Methods: Posts on each platform were searched from January 2017 through July 2022 using the terms “home birth” and “home vs. hospital birth”. Included posts were from the United States written in English, with at least 10 comments and 10 upvotes or likes. Up to five themes were collected per post or comment and were categorized as supportive, opposing, or neutral. The Institutional Review Board (IRB) determined that the project did not include human subjects.

Results: Collectively, 777 posts and 47,452 comments were evaluated for inclusion; 257 posts and 2,408 comments met the inclusion criteria for analysis. In posts, 69% supported, 20% opposed, and 11% were neutral toward home birth (n = 257). Similarly, in comments, 53% supported, 28% opposed, and 19% were neutral (n = 2,408). Supportive themes included concerns about the safety of hospital delivery and reassurance about home birth safety, enhanced patient control with home deliveries, positive personal stories reinforcing home birth, concerns about excessive interventions in hospital birth, and advice about preparing for home birth. Opposing themes included concerns about risks of home birth, negligence of those attempting it, reassurance that hospital birth does offer women control, greater financial costs of home birth and that medical interventions can be lifesaving.

Conclusion: These results can help physicians recognize some of the women’s concerns about hospital births and what information they may find on social media guiding them as they formulate their birth plans. Overall, this information helps with the goal of balancing patient safety with the need to respect patient autonomy.

## Introduction

From 2019 to 2020, the number of home births in the United States (US) increased by 20.2%; from 2020 to 2021, there was an additional increase of 12% [1,2]. At the end of 2021, approximately 1.41% of all births occurred at home [2]. The safety and cost-effectiveness of planned home births have been demonstrated among low-risk women with qualified midwives when they take place in the context of an integrated maternity care system [3-7]. However, in the US, multiple studies have shown that planned home births are associated with higher risks than hospital births. Perinatal deaths (a combination of fetal and neonatal deaths) at home were double those in hospital births and odds for neonatal seizures, and admission to neonatal intensive care units were higher with planned home births [8]. As a result of these poor outcomes, the American College of Obstetricians and Gynecologists (ACOG) recommends against home birth but supports discussing the potential risks and benefits of home births with interested and eligible candidates [9,10]. “Planned home birth”, defined as giving birth at home while still having prenatal care, testing, professional support, and, hopefully, backup plans for transfers, must be distinguished from “free birth”, which is the practice of giving birth with no medical assistance.

Further investigation is needed to understand why an increasing number of women are choosing planned home birth. Some of the increase in home births may result from increasing barriers to maternity care. Maternity care deserts are increasing - nearly seven million women in the US have little or no access to maternity care [11]. Women in these deserts may have no other realistic alternative to planned home birth. However, home births are also being selected by those who do have available hospital delivery services available. It is these women’s motivations that we sought to identify.

Reasons for choosing home birth have previously been examined through structured online surveys and a study of online responses to a mock news article [12-14]. These revealed that respondents favored home births because they previously had suffered a negative experience with hospital care or because they felt that home birth offered greater safety, avoidance of unnecessary medical interventions, and provided them more control and a comfortable environment [12-14]. One study of YouTube videos identified birth stories shared during the COVID-19 pandemic and categorized themes around their experiences; this included sense of loss, hospital experience, experience with healthcare professionals, and the choice of home birth over hospital birth [15].

However, there has not been an analysis of posts on social media platforms that allow for anonymous and spontaneous expressions, thus getting at many women’s unfiltered thoughts. Social media has become a primary way people in the US receive information on all topics, including healthcare [15-17]. Social media platforms are a means by which people can share their personal and emotional experiences of giving birth to influence the choices of others. We sought to study the posts and comments people made about home birth on two of the most widely used social media sites - Reddit and TikTok. Reddit is a prominent and influential platform that has 430 million monthly active users, with 48% of users being in the US, and over 138,000 communities called subreddits [18,19]. Users can be anonymous, giving many people a feeling of freedom of expression. While it has been difficult to collect demographic information of Reddit users due to anonymity, it has been reported that about 58% of users are between 18 and 34 years old, and 43% are female [19]. TikTok is a newer but increasingly popular social media platform with 150 million active users monthly in the US as of February 2023. It is reported that 85% of TikTok users are under 35 years old, and 61% identify as female [20,21].

The posts and comments were categorized into themes to help better understand women’s current thoughts and decisions regarding planned home births. Hopefully, this work will aid providers of obstetric care in tailoring their discussions of the risks and benefits of home births to be more current and relevant to their patients and to identify how to provide for those who may have to deliver at home.

## Materials and methods

Reddit and TikTok posts from January 2017 through July 2022 were reviewed using the search words “home birth” and “home vs. hospital birth”. The search terms “pro-home birth”, “community birth”, “midwife”, “out of hospital birth”, “at home birth”, “pregnancy home delivery”, and “natural home birth” were also used on Reddit. From the “home birth” search term, searches were made to further investigate additional related themes that were found. This included “home birth and covid”, “home birth and trans men”, and “home birth and birth trauma.” On Reddit, each search term was initially typed into the search bar, and resulting posts were organized by “relevance” for “all time”. Relevance in Reddit prioritizes the posts by the degree to which they relate to the search words. While Reddit does contain posts from all time, some were archived and did not populate older posts with a search in the main search bar. To access older posts, the search term “home birth” was put into a Google Search as “home birth reddit”. On Reddit, there are specific subreddits, or communities, organized by a specific theme. With both search terms, particular subreddits were commonly seen, so those terms were also searched in Reddit within those communities. These subreddits included r/homebirth, r/pregnant, r/BabyBumps, r/AskDocs, and r/medicine. On TikTok, each term was entered into the search bar, and results were viewed under the heading “video”. Videos were sorted by “relevance” rather than “most liked.” No filters isolating “liked videos” or “watched videos” were applied.

Inclusion criteria for posts were English language and having a minimum of 10 comments and 10 upvotes/likes. Exclusionary criteria for posts and comments included explicitly being from outside the US, not relating directly to home birth (e.g., reports about a reality show personality or social media influencer who happened to have a home birth), home births that were not planned, and posts about free birth, not home birth. There was no exclusion based on the gender of the poster. No personal identifying information was gathered.

Posts and comments were sequentially evaluated until thematic saturation was achieved. Once five comments on a post yielded no new themes, the investigator moved on to the next post. Saturation for posts within a search word was defined as finding no new themes in 10 consecutive posts, after which the process moved to a new search word [22]. All posts resulting from all search words were completely evaluated. For each post and each comment, up to five of the most significant themes were collected. Each extracted theme was categorized as being supportive, opposing, or neutral on home birth. Neutral themes were defined as not being fully in support of or against home birth where no firm position for either side was taken. In this study, results from both social media sites were combined to reflect information about a broader audience.

## Results

Only the search terms “home birth” and “home vs. hospital birth” yielded any posts. All the other search terms, including “pro-home birth”, “community birth”, “midwife”, “out of hospital birth”, “at home birth”, “pregnancy home delivery”, “natural home birth” and “home birth and birth trauma”, did not produce relevant results. In Reddit, results were found in all subreddits, except r/AskDocs for both search terms. Table 1 displays the percentage of posts analyzed by search word and subreddit.

**Table 1:** Subreddit sources of posts by search words in Reddit. The data have been represented as n, for the number of posts, and total n (%).

“Home Birth” (n)		“Home vs. Hospital Birth” (n)		Total n (%)
r/homebirth	20	r/homebirth	32	52 (37%)
r/BabyBumps	15	r/BabyBumps	9	24 (17%)
r/pregnant	13	r/pregnant	2	15 (11%)
r/medicine	3	r/medicine	3	6 (4%)
r/AskDocs	0	r/AskDocs	0	0 (0%)
Other	39	Other	6	45 (31%)

**Figure 1:**
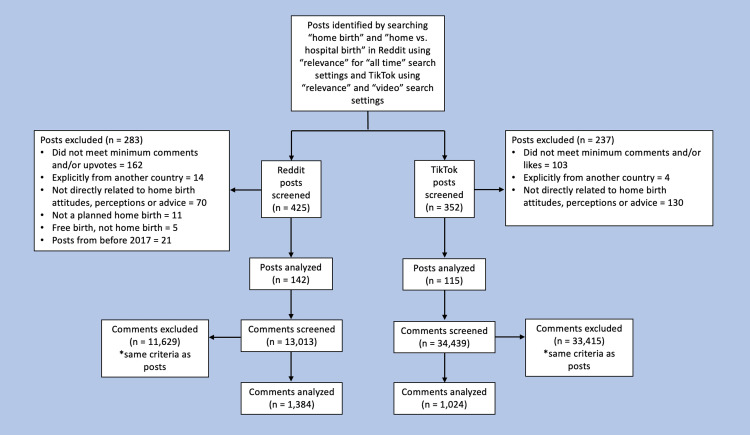
Flow diagram of home birth posts and comments included for analysis. The data have been represented as n, for the number of posts or comments.

Based on the remaining search terms, “home birth” and “home vs. hospital birth”, a total of 777 posts and 47,452 comments were evaluated for inclusion. From those, 257 posts and 2,408 comments met the criteria to analyze for thematic content (Figure 1).

Overall, 69% of posts were supportive of home birth; while 20% opposed home birth, and 11% were neutral (n = 257). In comments, 53% supported, 28% opposed, and 19% were neutral (n = 2,408). There was an abundance of themes found in these searches.

The major themes in support of home birth included the ability of the patient to control the birthing process, superior safety of the home, positive stories, avoiding excessive hospital interventions, and advice/support to those considering home birth (Table 2). Quotes exemplifying these themes are shown in Table 3.

**Table 2:** Number of comments and posts mentioning each supportive theme for reasons for choosing, and advice around, home birth. The data have been represented as n (%). The total exceeds 100% because multiple themes were extracted from each post or comment.

Supportive Themes	Posts (Total n = 178)	Comments (Total n = 1281)
Ability to control birth	79 (44%)	186 (15%)
Superior safety of home birth	52 (29%)	253 (20%)
Positive stories	44 (25%)	244 (19%)
Avoiding excessive intervention	31 (17%)	136 (11%)
Seeking advice/support	19 (11%)	110 (9%)

**Table 3:** Quotes from posters exemplifying themes in support of home birth. The data have been represented as quotes from posters on Reddit or TikTok.

Supportive Themes	Quotes from Posters
Ability to control birth	"For some people, it's not the 'financial' that they give birth at home. It's the comfort of home", "My nurse literally told me I was being too loud... in an unmedicated, induced, dry birth...", "but if you want people having hospital births shouldn't you be trying actively to make hospitals a more comfortable and patient-centered environment?".
Superior safety of the home birth	“It is true that doctors often bulldoze over women (even more so if you are of color) when it comes to their medical concerns”, “the doctor performed procedures without explaining them or getting my consent. My desires were ignored”, “Babies are safe at home births in all outcomes and also have an entirely different microbiome", "Hospitals notoriously sabotage normal birth".
Positive stories	"After a homebirth could never imagine birthing in a hospital again, magical experience we are incredible creatures", "We were able to show that homebirth is a powerful, beautiful experience".
Avoiding excessive intervention	"The cascade of interventions, dive labor into an emergency, that they then rescue you out of, and it creates a trauma bond of 'thank goodness I was in a hospital' ", "They coerced me into interventions I didn't want with fear and bullying."
Seeking advice/support	“I’ve been planning to have a home birth but as it gets closer I’m scared and anxious going into it”, “As a reader it's so disheartening to see post after post where women are told their body isn't capable of doing something it was literally made to do".
Birth trauma or sexual trauma	"I had a traumatic first delivery in a hospital that was traumatic solely because of the ridiculous amount of medicalization and violation that happened to me in the hospital. I never want to deliver in a hospital ever, ever again.”
Free birth	“Birth is overmedicalized but I would never have a free birth, that’s just reckless”.

The more frequent supporting theme was a greater sense of *control in the home*. The home was perceived to be a comfortable and relaxing place where women could deliver at their own pace and with their own preferences. Posters felt that they could effectively advocate for themselves more at home than in a hospital and believed that midwives at home gave more time and support to them than an obstetrician in a hospital would. A subset of the theme of control was a hyper-focus on their birth not deviating from the exact birth plan, which was especially reinforced by reading picture-perfect birth stories on social media. The *superior safety of the home* was also mentioned frequently. People had concerns about going into a hospital to give birth during COVID, but there was also a general fear of hospitals. Some had previous negative experiences with hospital births, while others had basic mistrust of all doctors or healthcare professionals. The home was often perceived as a safer place for people of color or people identifying as LGBTQ because of the potential bias they thought they would face in a hospital setting. Some women felt they were not asked to consent to any of the procedures in the hospital, and they were not listened to by their doctors. *Personal positive home birth stories *were also common. Some stated that they felt that there would be *excessive and unnecessary tests and procedures *performed during a hospital delivery. High rates of C-sections and concern about being coerced into having a surgical delivery when they felt it was not indicated led to the list of procedures they feared. Additionally, many people were asking for *advice *about home birth or for *support *going into it. Some advice women asked for was about how to handle the relationship tension with family and friends their decision was causing.

Some women had previous *birth trauma or sexual trauma* that pushed them more toward wanting a home birth. This theme was mentioned in seven posts and 20 comments on Reddit. Another minor theme, only found on Reddit, was confusion and frustration from supporters of home birth who were grouped together with people who choose *free birth*, even though they are very different experiences. This was mentioned in two posts and 22 comments.

Nearly one in five posts was against home birth. Table 4 summarizes the frequency of these themes, and Table 5 shows quotes exemplifying these themes.

**Table 4:** Numbers of posts and comments mentioning opposing themes for reasons and advice about not choosing home birth. The data have been represented as n (%). The total exceeds 100% because multiple themes were extracted from each post or comment.

Opposing Themes	Posts (Total n = 50)	Comments (Total n = 664)
Dangers of home birth/negligence of those choices	28 (56%)	261 (39%)
Superior safety of the hospital	24 (48%)	183 (28%)
Control over the process still possible	9 (18%)	78 (12%)
Cost savings	5 (10%)	37 (6%)
Interventions	4 (8%)	100 (15%)

**Table 5:** Quotes from posters exemplifying themes opposing home birth. The data have been represented as quotes from posters on Reddit or TikTok.

Opposing Themes	Quotes from Posters
Dangers of home birth/negligence of those choices	"Complications don’t only occur as a result of high-risk pregnancies. My wife had no risk factors and was a low-risk pregnancy...Medical best practice isn't determined by fair winds, but stormy seas”, "I immediately get anxiety for first time moms who don't know all the things that can go wrong at home", "Only in hindsight have I realized that my midwife only painted us the picture of the ideal successful homebirth. When it didn't go that way, it's like our experience didn't exist and we were left traumatized”.
Superior safety of the hospital	“I had a great hospital birth experience, my nurses had my back and I felt supported by my OB. When I started to have complications, I felt totally take care of”.
Control over the process still possible	"I used all midwives and requested an all women staff and minimal people in my suite. I labored in the shower, tub, got to be in any position I wanted...".
Cost savings	“It was the cost of home birth that made us choose the hospital”.
Interventions	“My baby was born with her cord wrapped around her neck and had swallowed meconium, I’m thankful for the interventions that saved her life”.

These posts describe the *dangers *perceived with home births and the *misinformation *they felt women were being given about the safety of home births. The dangers included concerns about a lack of training of many midwives, who often attend home births, and these midwives’ failure to recognize medical contraindications to home births. The unpredictability of medical emergencies occurring during labor and delivery even for low-risk patients was also mentioned in these entries. Posters expressed concern that women may not be aware that the home setting cannot adequately care for women experiencing such unpredictable complications. Another reason (*superior safety of the hospital*) stated was that the hospital is viewed as inherently *safer*, even during times of COVID. Women expressed concern that being outside a hospital when emergencies arise could significantly jeopardize their safety. People against home birth stated there is more *control *in the hospital than many pregnant people assume and that women should educate themselves and advocate for their own health in the hospital to maintain a desired sense of control. In contrast to citing *cost *as a reason to choose home birth, some mentioned that the out-of-pocket cost of midwives can be more expensive than a hospital birth. Many also viewed *interventions *and modern medicine as a good thing because it saves lives.

Some themes did not fit into either category and were classified as neutral (11% of posts overall, n = 257). This included people suggesting the use of birth centers or using a midwife and doula in a hospital as in-between options. Some people stated that there are obstetricians with more of a hands-off approach that supports low intervention in the hospital, so a provider can be found that will listen to patient desires while there is also increased safety. They stated physicians should work with patients to come to an agreement with them on what they desire during birth and should develop a birth plan for themselves. Some people also expressed that ultimately birthplace is the individual’s choice, so there is no right or wrong answer; it depends on the pregnant individual’s situation and what she wants to do. Quotes describing these themes are in Table 6.

**Table 6:** Quotes exemplifying themes neutral on home birth. The data have been represented as quotes from posters on Reddit or TikTok.

Neutral Themes	Quotes from Posters
Compromise	"The idea of a home birth sounds magical, I'd love to do it, but I think as close as I'll ever dare risk it is a birthing center very close to or attached to a hospital".
Individual’s choice	"Home birth is not for everyone, and I take issue with it being held up as this unrealistic standard of either 'you're a horrible mom for putting your child at risk' or 'you're a real mom ONLY IF YOU HOME BIRTH'".

Overall, there was an overlap in themes found between Reddit and TikTok; a majority were in support of home birth despite having generally different demographics. However, there were some key differences to point out. On TikTok, there were more mentions of cost as an important factor in deciding between home birth and hospital birth. TikTok also seemed to have fewer explicit charges of negligence mentioned for choosing home birth than on Reddit, although safety was still questioned. Although subreddits, including r/AskDocs and r/medicine, that provided professional advice were available, posts and comments were very infrequent on these sites.

## Discussion

The majority of posts and comments on these two popular social media platforms seem to support planned home births. Perhaps these favorable opinions are influenced by reports from other countries where out-of-hospital births, including planned home births by low-risk women, demonstrated safety [4,5]. In addition, the radical changes in obstetrical birthing experiences during the COVID pandemic may have influenced expectant women to choose home births. With the expansion of maternity care deserts across the US, many patients may have no realistic opportunity to have an in-hospital delivery.

Our findings on the popular social media platforms Reddit and TikTok generally confirm the findings of earlier survey work that, among women who favor home births, there are two primary reasons for this preference: control and safety [12]. First, posters feared hospital births because they perceived they would lose control of the birthing process - that they would not be allowed their idealized birth process and would be exposed to (unnecessary) medical interventions. Second, they preferred the home setting because they perceived it to be safer - they would avoid the dangers intrinsic to the hospital setting and have more comfort and personalized attention at home. Another important subgroup among those who supported home birth were those who had previously faced (or perceived they had faced) bias or trauma in healthcare settings, whose issues require additional attention [23,24]. Often on social media posts, it seemed as if women were seeking reassurance and support from their peers for their decision to have home birth, as well as advice on how to implement their plans and to gain approval from friends and family. The fact that individual stories posted were so positive should raise concerns about the unrealistic superiority of home births because readers are heavily influenced by social media stories [25-27]. Anecdotally, we noticed that women seemed to seek advice from peers more than going to community pages on social media that offered professional advice.

The disappointments and fears expressed by those who favored planned home birth - that they would not be able to experience the birth they dreamed of - may be at least partially addressed by increasing communications between patients and their clinicians, including a formal discussion/agreement about birth plans. Aragon et al. found that the process of developing a birth plan could be very educational; women learned about their options and hospital policy and were given a formal opportunity to address their concerns [28]. Ghahremani et al. highlighted the higher birth satisfaction of patients, who have such agreed-upon plans [29]. Ajayi et al. studied YouTube videos to document what problems birthing mothers faced with the experience to help clinicians improve patient satisfaction [15]. Grunebaum et al. offered a decision-aid checklist for patient counseling about out-of-hospital births, which included points about interventions, risks, and overall safety [30]. However, what was not mentioned in that checklist were the ideas of women seeking control, advice and support, and the influence of personal stories that were found through this social media search. We believe that our work amplifies the need for birth plan discussions to set realistic expectations and to enable shared decision-making that might make women feel they have a voice in advance and in real time.

The posts and comments from the minority who opposed home births emphasize the risks of home births that need to be remedied if patients in maternity care deserts are going to have safer home births. In particular, the uneven training of midwives attending home births in the US must be addressed. More than two-thirds of certified professional midwives (CPM) attending home births in the US do not meet minimum education standards, and at least 30% of those births are not low risk and do not meet clinical criteria set by ACOG and AAP [30,31]. The American College of Nurse Midwives lays out standards for education, which meet global guidelines from the International Confederation of Midwives, for Certified Nurse Midwives (CNM) and Certified Midwives (CM). This does not include CPM [32].

The strengths of this study included the fact that established rules and guidelines were followed for searching posts, including the use of thematic saturation, uniform search words, and inclusion and exclusion criteria. However, there are some limitations to this study. We were unable to exclude all posts and comments coming from other countries where home births are established and safer. This was a qualitative study with a degree of subjectivity and human error. Despite these limitations, our study provides important contributions directly from patient perspectives about attitudes and reasons for choosing home birth beyond just the ideas of medical interventions and safety.

## Conclusions

This study shows that women on two highly popular social media platforms have definite and disparate opinions about the safety of planned home births that are often at odds with professional guidelines in the US. As social media continues to grow as a resource, it is even more important now that there be effective communication between patients and their clinicians, specifically around forming a birth plan. This includes having a shared decision-making process where patient autonomy is upheld but also includes patient education about the risks and benefits of their options. The risks of home birth in the US still need to be addressed in the face of growing maternity care deserts, as promoting safety is the primary goal for all.
